# Dysfunction of Protein Quality Control in Parkinsonism–Dementia Complex of Guam

**DOI:** 10.3389/fneur.2018.00173

**Published:** 2018-03-20

**Authors:** Bert M. Verheijen, Kiyomitsu Oyanagi, Fred W. van Leeuwen

**Affiliations:** ^1^Department of Translational Neuroscience, Brain Center Rudolf Magnus, University Medical Center Utrecht, Utrecht University, Utrecht, Netherlands; ^2^Department of Neurology and Neurosurgery, Brain Center Rudolf Magnus, University Medical Center Utrecht, Utrecht University, Utrecht, Netherlands; ^3^Division of Neuropathology, Department of Brain Disease Research, Shinshu University School of Medicine, Nagano, Japan; ^4^Brain Research Laboratory, Hatsuishi Hospital, Chiba, Japan; ^5^Department of Neuroscience, Faculty of Health, Medicine and Life Sciences, Maastricht University, Maastricht, Netherlands

**Keywords:** Guam parkinsonism–dementia complex, mutant ubiquitin, protein quality control, protein aggregation, tau, neurofibrillary tangles, 43-kDa TAR DNA-binding protein, neuropathology

## Abstract

Guam parkinsonism–dementia complex (G-PDC) is an enigmatic neurodegenerative disease that is endemic to the Pacific island of Guam. G-PDC patients are clinically characterized by progressive cognitive impairment and parkinsonism. Neuropathologically, G-PDC is characterized by abundant neurofibrillary tangles, which are composed of hyperphosphorylated tau, marked deposition of 43-kDa TAR DNA-binding protein, and neuronal loss. Although both genetic and environmental factors have been implicated, the etiology and pathogenesis of G-PDC remain unknown. Recent neuropathological studies have provided new clues about the pathomechanisms involved in G-PDC. For example, deposition of abnormal components of the protein quality control system in brains of G-PDC patients indicates a role for proteostasis imbalance in the disease. This opens up promising avenues for new research on G-PDC and could have important implications for the study of other neurodegenerative disorders.

## Introduction

Guam parkinsonism–dementia complex (G-PDC) is a mysterious neurodegenerative disorder that afflicts the indigenous Chamorro people of Guam (Mariana Islands). It was first described in clinicopathological studies by Hirano et al. in 1961 (1, 2). Mental deterioration and extrapyramidal signs (rigidity, tremors, and bradykinesia) characterize G-PDC clinically (3). Neuropathologically, G-PDC is characterized by widespread neurofibrillary tangles (NFTs), consisting of highly phosphorylated tau, marked deposition of 43-kDa TAR DNA-binding protein (TDP-43) and neuronal loss (4).

A remarkably high incidence of amyotrophic lateral sclerosis (ALS) was previously found in the same Western Pacific focus of neurological disease (5–12). Because of co-occurrence and overlapping pathology, displaying features of classical ALS in combination with NFTs, it has been proposed that these cases of ALS and G-PDC are variations of the same disorder, i.e., ALS/PDC of Guam (known in Guam as *lytico-bodig*) (13, 14). However, ALS and G-PDC might be completely separate disease entities: NFTs have been reported to be a frequent background feature in the affected population, and, therefore, occurrence of ALS in Guamanians often results in mixed pathology (15–20).

Although several causes have been suggested for G-PDC, including genetics, infectious agents, mineral deficiencies, and environmental toxins, the etiology and pathogenesis of this disease remain unknown (21–26). Determining the cause(s) of G-PDC and the pathways that lead to neurodegeneration in G-PDC is of great interest, as this could lead to new insights into more common neurodegenerative diseases, such as Alzheimer’s disease (AD) and other tauopathies. Similarly, insights derived from studies on other neurodegenerative disorders may be helpful for understanding and revealing unique aspects of G-PDC. Here, we highlight some recent observations that open up new opportunities to investigate disease mechanisms in G-PDC.

## Neuropathological Features of G-PDC

The search for the cause of G-PDC has become increasingly challenging due to a notable decline in neurological disease incidence on the island in the last decades (27–31). The rapid Westernization of Guam may have contributed to this decrease. However, neuropathology has been found to be unaltered during this time (although the severity and distribution of pathological lesions may have changed to some extent) (32).

**Figure 1:**
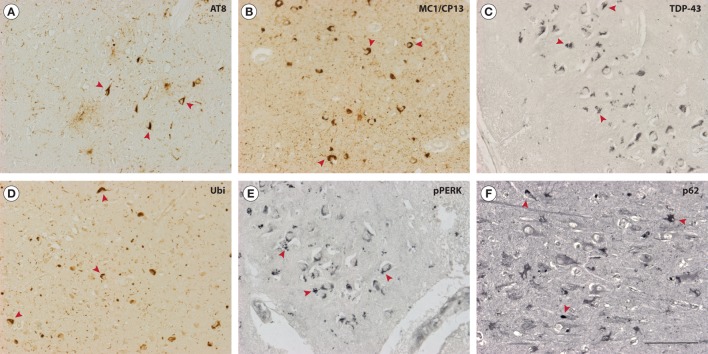
Neuropathological features of Guam parkinsonism–dementia complex (G-PDC). In the brains of G-PDC patients, various pathological protein aggregates can be found, e.g., neurofibrillary tangles (NFTs) (mouse anti-phosphorylated tau, AT8, 1:3,000, Innogenetics; mouse anti-MC1/CP13, 1:200, gift from Dr. P. Davies) **(A,B)** and 43-kDa TAR DNA-binding protein (TDP-43)-positive inclusions (mouse anti-TDP-43, 1:1,000, Abnova) **(C)**. Abnormal protein deposits are decorated with ubiquitin (rabbit anti-ubiquitin, Z0458, 1:3,000, DAKO) **(D)**. Immunoreactivity for phosphorylated pancreatic ER kinase (pPERK) indicates activation of the unfolded protein response and is associated with granulovacuolar degeneration (rabbit anti-pPERK, sc-32577, 1:400, Santa Cruz Biotechnology) **(E)**. Furthermore, the ubiquitin-binding protein p62 is deposited in G-PDC brains (rabbit anti-p62, 1:500, Biomol) **(F)**. Accumulation of p62 is a marker for the inhibition of autophagic flux. The photomicrographs in panels **(A–F)** show representative hippocampal sections from G-PDC brains. Arrowheads indicate distinct immunoreactive structures. Scale bar: 100 µm (Verheijen et al., unpublished data).

Neuropathologically, G-PDC is characterized by severe neuronal loss and abundant NFTs in different brain regions, including the temporal and frontal cortex, basal ganglia, thalamus, and brainstem (2, 17, 32, 33). NFTs (Figures 1A,B) are biochemically and ultrastructurally similar to those found in AD brains (34) and closely resemble those seen in frontotemporal dementia (FTD) tauopathies (35).

Besides frequent tangles, other pathological protein deposits have been identified in neurons of G-PDC brains. These include cytoplasmic TDP-43-containing aggregates (Figure 1C) and focal α-synuclein pathology (32, 36–40). This could hint at a role for these particular proteins in specific disease processes in G-PDC. For example, abnormal aggregation of the RNA-binding protein TDP-43 may be linked to defective RNA processing (e.g., pre-mRNA splicing) and several other cellular perturbations, like disrupted nucleocytoplasmic transport, mitochondrial dysfunction, and inhibition of endocytosis (41–46). Amyloid-β (Aβ) deposits were initially reported to be rare or absent in G-PDC (47) but were later found to be present by several authors (32, 48–51). Intracellular inclusions are often ubiquitinated (32) (Figure 1D).

It is interesting to point out that glial cells, i.e., astrocytes and oligodendroglia, also contain pathological inclusions (tau, TDP-43) in G-PDC and that these cells may play a significant role in G-PDC pathogenesis (32, 52). Extracellular NFTs are associated with reactive microglia (53, 54).

Importantly, findings related to multiple proteinopathy have been complemented by the detection of molecular markers that link G-PDC to certain protein homeostasis (proteostasis) pathways. We have recently found some evidence for activation of the unfolded protein response (UPR) in G-PDC brain (Verheijen et al., unpublished data). The UPR is an adaptive signaling cascade that is triggered by endoplasmic reticulum (ER) stress and is associated with abnormal protein aggregation and neurodegeneration (55, 56). Phosphorylation of pancreatic ER kinase, an ER transmembrane protein and ER stress sensor, plays an important role in the initiation and regulation of the UPR (Figure 1E). The primary goal of the UPR is to restore proteostasis in the ER via translational block and activation of ER stress responsive genes, but extensive or prolonged ER stress and UPR activation in disease can turn the UPR maladaptive. In addition, the autophagy substrate p62/SQSTM1 accumulates in G-PDC brains (Figure 1F), which could indicate compromised autophagy-mediated degradation (57, 58).

Together, some of these pathological characteristics suggest proteostasis network dysfunction in G-PDC. Disruption of the intracellular protein degradation machinery and proteostasis collapse has been associated with many neurodegenerative diseases, and it will be interesting to see if, and to what extent, protein degradation pathways are affected in G-PDC (59, 60). Failure of protein quality control (PQC) mechanisms to maintain proteostasis may represent a key pathogenic mechanism in G-PDC.

## Mutant Ubiquitin and Impaired Proteolysis in G-PDC

Multiple neuropathological observations imply impaired PQC in G-PDC. In a recent study, abnormal components of the ubiquitin-proteasome pathway (UPP), i.e., frameshift mutants of ubiquitin-B (UBB^+1^), have been found to accumulate in G-PDC brains (61).

**Figure 2:**
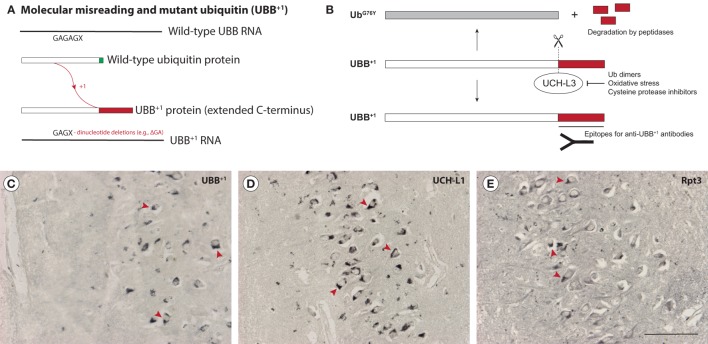
Accumulation of mutant ubiquitin (UBB^+1^) in Guam parkinsonism-dementia complex (G-PDC). UBB^+1^ is a frameshift mutant of ubiquitin that is generated through “molecular misreading,” a form of transcriptional mutagenesis that introduces dinucleotide deletions (; ΔGA or ΔGU) in or near GAGAG motifs in mRNA **(A)**. UBB^+1^ contains an extended C-terminal tail that can be recognized by anti-UBB^+1^ antibodies. Deubiquitinating enzymes (DUBs), i.e., ubiquitin C-terminal hydrolase L3 (UCH-L3), can cleave this abnormal C-terminal domain, destroying the epitope. However, inhibition of DUBs, e.g., by oxidative stress conditions, prevents this cleavage **(B)**. Accumulation of UBB^+1^ (rabbit anti-UBB^+1^, Ubi2A, 1:400, Dr. F. W. van Leeuwen) **(C)** and specific ubiquitin-proteasome pathway components, i.e., the DUB ubiquitin C-terminal hydrolase L1 (UCH-L1) (rabbit anti-UCH-L1, 1:500, Biomol) **(D)** and the proteasomal ATPase subunit Rpt3 (rabbit anti-Rpt3, 1:400, Biomol) **(E)** can be observed in G-PDC patient brains (hippocampal sections), which strongly suggests proteostasis breakdown. UBB^+1^ is not present in young control brains (non-Guamanian cases). Arrowheads indicate various immunoreactive structures. Scale bar: 100 µm (Verheijen et al., unpublished data).

The UPP is a major mechanism for the clearance of (abnormal) proteins in cells and impairment of the UPP has been reported to occur during neurodegeneration (62–65). UBB^+1^ is a dose-dependent inhibitor of the UPP that is thought to be generated through “molecular misreading,” a poorly understood process that introduces mutations (e.g., ΔGA or ΔGU dinucleotide deletions) not present in DNA into mRNA, resulting in the generation of aberrant proteins (66–68) (Figure 2A). The mutant protein lacks a C-terminal glycine residue (G76, which is replaced by a 20-amino acid extension in UBB^+1^) that is necessary to ubiquitinate other proteins, but can still be ubiquitinated itself. The abnormal C-terminal domain of UBB^+1^ can be recognized by UBB^+1^ antibodies (69) (Figures 2B,C).

UBB^+1^ accumulates in several neurodegenerative diseases other than G-PDC, including tauopathies (e.g., AD) and polyglutamine (polyQ) diseases (e.g., Huntington’s disease) and has been shown to be detrimental to neurons (66, 70–73). The mechanisms by which UBB^+1^ exerts its effects on the UPP and neuronal function are not known exactly, but it has been demonstrated that UBB^+1^ is an inhibitor of deubiquitinating enzymes (DUBs) (74), and that it can affect mitochondrial function (75). UBB^+1^ expression in primary neurons causes neuritic beading of mitochondria in association with neuronal degeneration, presumably due to impaired axonal transport (76). Transgenic expression of UBB^+1^ in mice results in contextual memory deficits and central breathing dysfunction, which are consistent with neurodegenerative disease (77, 78). Also, UBB^+1^ was shown to increase mutant protein load in a mouse model for familial encephalopathy with neuroserpin inclusion bodies (FENIB), by impairing ER-associated degradation (79). UBB^+1^ might act as a modifier of the aggregation and cytotoxicity of other aggregation-prone proteins, such as prion protein and disease-associated huntingtin (80).

Dissecting the precise role of UBB^+1^ in neurodegeneration is complicated by its dual role as both substrate and inhibitor of the UPP: at low concentrations, UBB^+1^ is degraded via the ubiquitin-fusion degradation pathway, but at high concentrations it is a potent inhibitor of the UPP (81–85). In addition, UBB^+1^ can induce cytoprotective programs, like chaperone expression (86), which may compensate for diminished UPP function. Overexpression of UBB^+1^ in AD transgenic mice resulted in an unexpected decrease in Aβ plaque load (87), which could suggest that accumulation of UBB^+1^ is actually part of a cellular protective response. It is tempting to speculate that UBB^+1^ can interfere with non-degradation-based PQC processes like misfolding-associated protein secretion (MAPS) as well, because MAPS enables PQC when conventional proteasomal degradation is impaired (88). Recent progress in identifying and understanding the roles of different ubiquitin chain topologies potentially adds another layer of complexity to the study of UBB^+1^, because different ubiquitin chain linkages on UBB^+1^ could change its properties (89–91). The E3 ubiquitin ligases and DUBs that regulate UBB^+1^ ubiquitination in neurons also remain to be identified (73).

In G-PDC, UBB^+1^ deposits are not exclusively located in neurons, but are also present in glial cells (61). Such glial deposits have previously been observed in progressive supranuclear palsy (71), a disease that shares some similarities with G-PDC (23, 92, 93). The role of the UPP in glia with regard to neurodegenerative disease has been somewhat neglected (94), and it would be interesting to find out how UBB^+1^ impacts neuroglia. In cultured astrocytes, UBB^+1^ expression seems to confer protective effects via various mechanisms, ranging from regulation of pro-inflammatory signaling to altering mitochondrial dynamics (95, 96). Thus, UBB^+1^ and UPP inhibition may elicit cell type-specific responses in the context of disease.

In addition to UBB^+1^, several other proteins related to the UPP were found to be present in G-PDC aggregates, i.e., the DUB ubiquitin C-terminal hydrolase L1 (UCH-L1) (Figure 2D) and the proteasomal ATPase subunit Rpt3 (Figure 2E) (61). Accumulation of these UPP components has been associated with neuropathology in earlier studies (97, 98). Mutations in UCH-L1 lead to motor dysfunction in patients (99, 100) and mice lacking functional UCH-L1 show neurodegeneration (101). Strikingly, motor neuron-specific knockout of Rpt3 results in an ALS-like phenotype in mice (102).

Based on these findings, we conclude that the UPP is disturbed in G-PDC. The definite roles of UBB^+1^, different UPP components, and PQC mechanisms in G-PDC remain to be determined.

## Proteostasis Imbalance and Neurodegeneration in G-PDC

Accumulation of (abnormal) PQC components in G-PDC brains could be an important clue as to why disease-associated proteins aggregate and neurons degenerate in G-PDC. Impaired PQC and several neurodegenerative diseases have been causally linked through genetic analyses of familial cases [e.g., in familial Parkinson’s disease (PD) (103)], demonstrating that PQC dysfunction can be a disease-initiating factor. Moreover, pharmacological inhibition of PQC via injection of proteasome inhibitors causes parkinsonian features in rats (104). Age-related decline in PQC function might (in part) explain the loss of proteome integrity associated with age-related neurodegenerative disorders, including G-PDC (105, 106).

Detailed examination of the PQC system and UBB^+1^ in the context of the multiproteinopathy that characterizes G-PDC, in different experimental models (e.g., *in vitro* neuronal cell culture and experimental animal models), will likely increase our knowledge of the timing (early vs. late) and relative importance of different disease processes. We take the view that mechanistic studies in such experimental model systems, and validating new findings against the reality of human tissues (neuropathology), will be a powerful approach for making discoveries. It is well recognized that abnormal PQC is implicated in AD, ALS(-FTD), PD, and other neurodegenerative disorders. Because this is a quite recent finding for G-PDC, insights derived from studies on other neurodegenerative diseases will be valuable to help understanding G-PDC, including its unique characteristics.

The cellular mechanisms that control proteostasis are not limited to protein degradation by the UPP, but also involve protein synthesis, folding, trafficking, and other (extracellular) clearance routes (107). PQC critically depends on additional cellular machinery, such as molecular chaperones (108, 109), and is often associated with specific cellular compartments/organelles (e.g., ER, mitochondria). Crosstalk within the proteostasis network and interactions between disease-related proteins and various disease mechanisms (e.g., DNA damage, defective RNA processing, ER and mitochondrial dysfunction, nitrative and oxidative stress, cytoskeletal defects, Golgi fragmentation, abnormal stress granule formation, prion-like mechanisms, neuroinflammation, synaptic malfunction, disrupted membrane trafficking, and excitotoxicity) can determine whether a neuron will degrade (toxic protein aggregates) or degenerate. It should be mentioned that protein aggregation itself, even though it reflects failure of PQC to get rid of potentially harmful proteins, need not be pathogenic and in some cases may even be protective (110).

Ultimately, new mechanistic insights may result in the identification of therapeutic targets that prevent or slow down neurodegeneration. If G-PDC and other neurological disorders converge on the dysregulation of proteostasis as a common underlying mechanism of pathogenesis, exploring PQC pathways to restore proteostasis could be a promising strategy to find therapies for multiple neurodegenerative diseases. Current advances include the use of UPP activation to remove and stop propagation of pathogenic protein species (111–113). Other targets, such as chaperones and autophagy, are also being explored (58, 109, 114). However, different proteins, in different protein states, can be cleared via separate pathways. Ideally, a proteostasis “reset,” rebalancing the entire proteostasis network in proteotoxically stressed neurons, would be desirable, because this could counteract many defects at the same time. It is unknown whether such a reset switch exists in human neurons, but some cells appear to exhibit a remarkable capacity to reestablish proteostasis (e.g., via lysosome acidification and metabolic shift in *C. elegans* germ lineage, “exopher” formation in *C. elegans* neurons and during differentiation of mouse embryonic stem cells) (115–117). Combinatorial therapies are likely to be required to generate robust treatments that effectively restore homeostasis in human somatic cells in such a way. The diverse pathology of G-PDC provides an excellent paradigm for interrogating interactions between multiple pathogenic proteins, the proteostasis network and different mechanisms of neurodegeneration.

## Future Directions

Novel neuropathological findings in G-PDC stress the importance of utilizing pathway-specific markers to unravel disease mechanisms. While determining the (possibly multifactorial) cause of G-PDC, perhaps through new genetic and/or exposome (i.e., the totality of all environmental exposures) studies in the geographical isolate, remains a major outstanding problem, more studies into the pathomechanisms underlying neurodegeneration in G-PDC are warranted. Applying insights derived from studies on other neurological diseases that share molecular neuropathology, including work on postmortem human brains and various experimental model systems, will help to identify critical disease mechanisms. Investigating the interplay between G-PDC-associated pathological proteins and specific cellular/molecular pathways (e.g., PQC pathways), using relevant experimental models (e.g., patient induced pluripotent stem cell-derived neurons), will be an exciting and challenging task for the future. Such studies have also been initiated on the Kii peninsula of Japan, another hyperendemic focus of ALS/PDC (118–120) (Verheijen et al., under investigation). The resulting insights could improve our understanding of other neurodegenerative diseases and might result in the identification of new biomarkers or therapeutic targets. It will be important for future studies to replicate findings in other populations with increased prevalence of ALS/PDC (121, 122).

## Some Open Questions

- Is impairment of the PQC system an important pathogenic mechanism in G-PDC?
- Does UBB^+1^ modify the cytotoxicity of aggregation-prone proteins, like tau and TDP-43?
- What is the role of glia in G-PDC? What are the effects of UBB^+1^ on glial cells?
- Are UBB^+1^ and PQC proteins also deposited in diseases that are similar to G-PDC, e.g., Kii ALS/PDC?
- What are the roles of the UPR and other adaptive stress response pathways in G-PDC?
- Is accumulation of UBB^+1^ and PQC dysfunction an early or late event in G-PDC? Is it a cause or consequence (or both) of disease?
- How can new insights into pathogenic mechanisms of G-PDC be used to advance the understanding and treatment of other neurodegenerative diseases? E.g., can UBB^+1^ and particular PQC components be used as therapeutic targets in neurodegenerative disorders?

## Author Contributions

All authors listed have made a substantial, direct, and intellectual contribution to the work and approved it for publication.

## Conflict of Interest Statement

The authors declare that the research was conducted in the absence of any commercial or financial relationships that could be construed as a potential conflict of interest.

## References

[1] Hirano A Kurland LT Krooth RS Lessell S Parkinsonism-dementia complex, an endemic disease on the island of Guam. I. Clinical features. Brain (1961) 84:642–61. 10.1093/brain/84.4.642 13907609

[2] Hirano A Malamud N Kurland LT Parkinsonism-dementia complex, an endemic disease on the island of Guam. II. Pathological features. Brain (1961) 84:662–79. 10.1093/brain/84.4.662 13907610

[3] Murakami N. Parkinsonism-dementia complex on Guam – overview of clinical aspects. J Neurol (1999) 246(Suppl 2):II16–8. 10.1007/BF03161077 10525998

[4] Oyanagi K Hashimoto T Yamazaki M Parkinsonism–dementia complex of Guam. In: Dickson DW Weller RO, editors. Neurodegeneration: The Molecular Pathology of Dementia and Movement Disorders. Oxford, UK: Wiley-Blackwell (2011). p. 171–8.

[5] Zimmerman H Progress Report of Work in the Laboratory of Pathology during May 1945. Guam. Washington, DC: US Naval Medical Research (1945). Unit Number 2, 1 June.

[6] Koerner DR Amyotrophic lateral sclerosis on Guam: a clinical study and review of the literature. Ann Intern Med (1952) 37:1204–20. 10.7326/0003-4819-37-6-1204 13008298

[7] Arnold A Edgren DC Palladino VS Amyotrophic lateral sclerosis; fifty cases observed on Guam. J Nerv Ment Dis (1953) 117:135–9. 10.1097/00005053-195302000-00005 13061952

[8] Tillema S Wijnberg CJ Endemic amyotrophic lateral sclerosis on Guam; epidemiological data; a preliminary report. Doc Med Geogr Trop (1953) 5:366–70. 13151035

[9] Kurland LT Mulder DW Epidemiologic investigations of amyotrophic lateral sclerosis. 1. Preliminary report on geographic distribution, with special reference to the Mariana Islands, including clinical and pathologic observations. Neurology (1954) 4:355–78. 10.1212/WNL.4.5.355 13185376

[10] Mulder DW Kurland LT Iriarte LL Neurologic diseases on the island of Guam. U S Armed Forces Med J (1954) 5:1724–39. 13217119

[11] Kurland LT Mulder DW Epidemiologic investigations of amyotrophic lateral sclerosis. 2. Familial aggregations indicative of dominant inheritance. I. Neurology (1955) 5:182–96. 10.1212/WNL.5.3.182 14356347

[12] Kurland LT Mulder DW Epidemiologic investigations of amyotrophic lateral sclerosis. 2. Familial aggregations indicative of dominant inheritance. II. Neurology (1955) 5:249–68. 10.1212/WNL.5.3.182 14370376

[13] Elizan TS Hirano A Abrams BM Need RL Van Nuis C Kurland LT Amyotrophic lateral sclerosis and parkinsonism-dementia complex of Guam. Neurological reevaluation. Arch Neurol (1966) 14:356–68. 10.1001/archneur.1966.00470100012002 5906460

[14] Hirano A Malamud N Elizan TS Kurland LT Amyotrophic lateral sclerosis and Parkinsonism-dementia complex on Guam. Further pathologic studies. Arch Neurol (1966) 15:35–51. 10.1001/archneur.1966.00470130039004 5939450

[15] Anderson FH Richardson EP Okazaki H Brody JA. Neurofibrillary degeneration on Guam: frequency in Chamorros and non Chamorros with no known neurological disease. Brain (1979) 102:65–77. 10.1093/brain/102.1.65 427533

[16] Chen L. Neurofibrillary change on Guam. Arch Neurol (1981) 38:16–8. 10.1001/archneur.1981.00510010042006 7458717

[17] Oyanagi K Makifuchi T Ohtoh T Chen KM van der Schaaf T Gajdusek DC Amyotrophic lateral sclerosis of Guam: the nature of the neuropathological findings. Acta Neuropathol (1994) 88:405–12. 10.1007/BF00389491 7847068

[18] Oyanagi K Wada M. Neuropathology of parkinsonism-dementia complex and amyotrophic lateral sclerosis of Guam: an update. J Neurol (1999) 246(Suppl 2):II19–27. 10.1007/BF03161078 10525999

[19] Morris HR Al-Sarraj S Schwab C Gwinn-Hardy K Perez-Tur J Wood NW A clinical and pathological study of motor neurone disease on Guam. Brain (2001) 124:2215–22. 10.1093/brain/124.11.2215 11673323

[20] Perl DP Hof PR Purohit DP Loerzel AJ Kakulas BA. Hippocampal and entorhinal cortex neurofibrillary tangle formation in Guamanian Chamorros free of overt neurologic dysfunction. J Neuropathol Exp Neurol (2003) 62:381–8. 10.1093/jnen/62.4.381 12722830

[21] Perl DP Gajdusek DC Garruto RM Yanagihara RT Gibbs CJ. Intraneuronal aluminum accumulation in amyotrophic lateral sclerosis and Parkinsonism-dementia of Guam. Science (1982) 217:1053–5. 10.1126/science.7112111 7112111

[22] Spencer PS Nunn PB Hugon J Ludolph AC Ross SM Roy DN Guam amyotrophic lateral sclerosis-parkinsonism-dementia linked to a plant excitant neurotoxin. Science (1987) 237:517–22. 10.1126/science.3603037 3603037

[23] Steele JC. Parkinsonism-dementia complex of Guam. Mov Disord (2005) 20(Suppl 12):S99–107. 10.1002/mds.20547 16092098

[24] Steele JC Guella I Szu-Tu C Lin MK Thompson C Evans DM Defining neurodegeneration on Guam by targeted genomic sequencing. Ann Neurol (2015) 77:458–68. 10.1002/ana.24346 25558820

[25] Steele JC Wresch R Hanlon SD Keystone J Ben-Shlomo Y A unique retinal epitheliopathy is associated with amyotrophic lateral sclerosis/Parkinsonism-Dementia complex of Guam. Mov Disord (2015) 30:1271–5. 10.1002/mds.26264 26153661

[26] Cox PA Davis DA Mash DC Metcalf JS Banack SA. Dietary exposure to an environmental toxin triggers neurofibrillary tangles and amyloid deposits in the brain. Proc Biol Sci (2016) 283:20152397. 10.1098/rspb.2015.2397 26791617 PMC4795023

[27] Garruto RM Yanagihara R Gajdusek DC. Disappearance of high-incidence amyotrophic lateral sclerosis and parkinsonism-dementia on Guam. Neurology (1985) 35:193–8. 10.1212/WNL.35.2.193 3969206

[28] Wiederholt WC. Neuroepidemiologic research initiatives on Guam: past and present. Neuroepidemiology (1999) 18:279–91. 10.1159/000026223 10545780

[29] Galasko D Salmon DP Craig U-K Thal LJ Schellenberg G Wiederholt W. Clinical features and changing patterns of neurodegenerative disorders on Guam, 1997–2000. Neurology (2002) 58:90–7. 10.1212/WNL.58.1.90 11781411

[30] Plato CC Galasko D Garruto RM Plato M Gamst A Craig U-K ALS and PDC of Guam: forty-year follow-up. Neurology (2002) 58:765–73. 10.1212/WNL.58.5.765 11889241

[31] Plato CC Garruto RM Galasko D Craig U-K Plato M Gamst A Amyotrophic lateral sclerosis and parkinsonism-dementia complex of Guam: changing incidence rates during the past 60 years. Am J Epidemiol (2003) 157:149–57. 10.1093/aje/kwf175 12522022

[32] Miklossy J Steele JC Yu S McCall S Sandberg G McGeer EG Enduring involvement of tau, beta-amyloid, alpha-synuclein, ubiquitin and TDP-43 pathology in the amyotrophic lateral sclerosis/parkinsonism-dementia complex of Guam (ALS/PDC). Acta Neuropathol (2008) 116:625–37. 10.1007/s00401-008-0439-2 18843496

[33] Nakano I Hirano A. Neuron loss in the nucleus basalis of Meynert in parkinsonism-dementia complex of Guam. Ann Neurol (1983) 13:87–91. 10.1002/ana.410130118 6830170

[34] Buée-Scherrer V Buée L Hof PR Leveugle B Gilles C Loerzel AJ Neurofibrillary degeneration in amyotrophic lateral sclerosis/parkinsonism-dementia complex of Guam. Immunochemical characterization of tau proteins. Am J Pathol (1995) 146:924–32. 7717459 PMC1869250

[35] Winton MJ Joyce S Zhukareva V Practico D Perl DP Galasko D Characterization of tau pathologies in gray and white matter of Guam parkinsonism-dementia complex. Acta Neuropathol (2006) 111:401–12. 10.1007/s00401-006-0053-0 16609851

[36] Yamazaki M Arai Y Baba M Iwatsubo T Mori O Katayama Y Alpha-synuclein inclusions in amygdala in the brains of patients with the parkinsonism-dementia complex of Guam. J Neuropathol Exp Neurol (2000) 59:585–91. 10.1093/jnen/59.7.585 10901229

[37] Forman MS Schmidt ML Kasturi S Perl DP Lee VM-Y Trojanowski JQ. Tau and alpha-synuclein pathology in amygdala of Parkinsonism-dementia complex patients of Guam. Am J Pathol (2002) 160:1725–31. 10.1016/S0002-9440(10)61119-4 12000724 PMC1850878

[38] Sebeo J Hof PR Perl DP. Occurrence of alpha-synuclein pathology in the cerebellum of Guamanian patients with parkinsonism-dementia complex. Acta Neuropathol (2004) 107:497–503. 10.1007/s00401-004-0840-4 15024581

[39] Hasegawa M Arai T Akiyama H Nonaka T Mori H Hashimoto T TDP-43 is deposited in the Guam parkinsonism-dementia complex brains. Brain (2007) 130:1386–94. 10.1093/brain/awm065 17439983

[40] Geser F Winton MJ Kwong LK Xu Y Xie SX Igaz LM Pathological TDP-43 in parkinsonism-dementia complex and amyotrophic lateral sclerosis of Guam. Acta Neuropathol (2008) 115:133–45. 10.1007/s00401-007-0257-y 17713769

[41] Lagier-Tourenne C Polymenidou M Cleveland DW. TDP-43 and FUS/TLS: emerging roles in RNA processing and neurodegeneration. Hum Mol Genet (2010) 19:R46–64. 10.1093/hmg/ddq137 20400460 PMC3167692

[42] Braun RJ Sommer C Carmona-Gutierrez D Khoury CM Ring J Büttner S Neurotoxic 43-kDa TAR DNA-binding protein (TDP-43) triggers mitochondrion-dependent programmed cell death in yeast. J Biol Chem (2011) 286:19958–72. 10.1074/jbc.M110.194852 21471218 PMC3103370

[43] Scotti MM Swanson MS RNA mis-splicing in disease. Nat Rev Genet (2016) 17:19–32. 10.1038/nrg.2015.3 26593421 PMC5993438

[44] Liu G Coyne AN Pei F Vaughan S Chaung M Zarnescu DC Endocytosis regulates TDP-43 toxicity and turnover. Nat Commun (2017) 8:2092. 10.1038/s41467-017-02017-x 29233983 PMC5727062

[45] Chou C-C Zhang Y Umoh ME Vaughan SW Lorenzini I Liu F TDP-43 pathology disrupts nuclear pore complexes and nucleocytoplasmic transport in ALS/FTD. Nat Neurosci (2018) 79:416. 10.1038/s41593-017-0047-3 29311743 PMC5800968

[46] Leibiger C Deisel J Aufschnaiter A Ambros S Tereshchenko M Verheijen BM TDP-43 controls lysosomal pathways thereby determining its own clearance and cytotoxicity. Hum Mol Genet (2018). 10.1093/hmg/ddy066 29474575

[47] Gentleman SM Perl D Allsop D Clinton J Royston MC Roberts GW Beta (A4)-amyloid protein and parkinsonian dementia complex of Guam. Lancet (1991) 337:55–6. 10.1016/0140-6736(91)93378-M 1670679

[48] Ito H Hirano H Yen SH Kato S. Demonstration of beta amyloid protein-containing neurofibrillary tangles in parkinsonism-dementia complex on Guam. Neuropathol Appl Neurobiol (1991) 17:365–73. 10.1111/j.1365-2990.1991.tb00736.x 1758569

[49] Guiroy DC Mellini M Miyazaki M Hilbich C Safar J Garruto RM Neurofibrillary tangles of Guamanian amyotrophic lateral sclerosis, parkinsonism-dementia and neurologically normal Guamanians contain a 4- to 4.5-kilodalton protein which is immunoreactive to anti-amyloid beta/A4-protein antibodies. Acta Neuropathol (1993) 86:265–74. 10.1007/BF00304141 8213085

[50] Schwab C Steele JC Akiyama H McGeer EG McGeer PL. Relationship of amyloid beta/A4 protein to the neurofibrillary tangles in Guamanian parkinsonism-dementia. Acta Neuropathol (1995) 90:287–98. 10.1007/BF00296513 8525803

[51] Schmidt ML Lee VM Saido T Perl D Schuck T Iwatsubo T Amyloid plaques in Guam amyotrophic lateral sclerosis/parkinsonism-dementia complex contain species of A beta similar to those found in the amyloid plaques of Alzheimer’s disease and pathological aging. Acta Neuropathol (1998) 95:117–22. 10.1007/s004010050774 9498044

[52] Oyanagi K Makifuchi T Ohtoh T Chen K-M Gajdusek DC Chase TN. Distinct pathological features of the Gallyas- and tau-positive glia in the parkinsonism-dementia complex and amyotrophic lateral sclerosis of Guam. J Neuropathol Exp Neurol (1997) 56:308–16. 10.1097/00005072-199703000-00010 9056545

[53] Schwab C Steele JC McGeer PL. Neurofibrillary tangles of Guam parkinson-dementia are associated with reactive microglia and complement proteins. Brain Res (1996) 707:196–205. 10.1016/0006-8993(95)01257-5 8919296

[54] McGeer PL Schwab C McGeer EG Haddock RL Steele JC. Familial nature and continuing morbidity of the amyotrophic lateral sclerosis-parkinsonism dementia complex of Guam. Neurology (1997) 49:400–9. 10.1212/WNL.49.2.400 9270568

[55] Scheper W Hoozemans JJM. The unfolded protein response in neurodegenerative diseases: a neuropathological perspective. Acta Neuropathol (2015) 130:315–31. 10.1007/s00401-015-1462-8 26210990 PMC4541706

[56] Hetz C Saxena S. ER stress and the unfolded protein response in neurodegeneration. Nat Rev Neurol (2017) 13:477–91. 10.1038/nrneurol.2017.99 28731040

[57] Menzies FM Fleming A Rubinsztein DC. Compromised autophagy and neurodegenerative diseases. Nat Rev Neurosci (2015) 16:345–57. 10.1038/nrn3961 25991442

[58] Menzies FM Fleming A Caricasole A Bento CF Andrews SP Ashkenazi A Autophagy and neurodegeneration: pathogenic mechanisms and therapeutic opportunities. Neuron (2017) 93:1015–34. 10.1016/j.neuron.2017.01.022 28279350

[59] Rubinsztein DC. The roles of intracellular protein-degradation pathways in neurodegeneration. Nature (2006) 443:780–6. 10.1038/nature05291 17051204

[60] Hipp MS Park S-H Hartl FU. Proteostasis impairment in protein-misfolding and -aggregation diseases. Trends Cell Biol (2014) 24:506–14. 10.1016/j.tcb.2014.05.003 24946960

[61] Verheijen BM Hashimoto T Oyanagi K van Leeuwen FW Deposition of mutant ubiquitin in parkinsonism-dementia complex of Guam. Acta Neuropathol Commun (2017) 5:82 10.1186/s40478-017-0490-0 29122008 PMC5679492

[62] Ciechanover A Brundin P. The ubiquitin proteasome system in neurodegenerative diseases: sometimes the chicken, sometimes the egg. Neuron (2003) 40:427–46. 10.1016/S0896-6273(03)00606-8 14556719

[63] Goldberg AL. Protein degradation and protection against misfolded or damaged proteins. Nature (2003) 426:895–9. 10.1038/nature02263 14685250

[64] Lehman NL. The ubiquitin proteasome system in neuropathology. Acta Neuropathol (2009) 118:329–47. 10.1007/s00401-009-0560-x 19597829 PMC2716447

[65] Dennissen FJA Kholod N van Leeuwen FW. The ubiquitin proteasome system in neurodegenerative diseases: culprit, accomplice or victim? Prog Neurobiol (2012) 96:190–207. 10.1016/j.pneurobio.2012.01.003 22270043

[66] van Leeuwen FW de Kleijn DPV van den Hurk HH Neubauer A Sonnemans MAF Sluijs JA Frameshift mutants of beta amyloid precursor protein and ubiquitin-B in Alzheimer’s and down patients. Science (1998) 279:242–7. 10.1126/science.279.5348.242 9422699

[67] van Leeuwen FW Burbach JP Hol EM. Mutations in RNA: a first example of molecular misreading in Alzheimer’s disease. Trends Neurosci (1998) 21:331–5. 10.1016/S0166-2236(98)01280-6 9720597

[68] van Leeuwen FW Fischer DF Kamel D Sluijs JA Sonnemans MAF Benne R Molecular misreading: a new type of transcript mutation expressed during aging. Neurobiol Aging (2000) 21:879–91. 10.1016/S0197-4580(00)00151-2 11124436

[69] Dennissen FJA Kholod N Hermes DJHP Kemmerling N Steinbusch HWM Dantuma NP Mutant ubiquitin (UBB^+1^) associated with neurodegenerative disorders is hydrolyzed by ubiquitin C-terminal hydrolase L3 (UCH-L3). FEBS Lett (2011) 585:2568–74. 10.1016/j.febslet.2011.06.037 21762696

[70] de Vrij FM Sluijs JA Gregori L Fischer DF Hermens WT Goldgaber D Mutant ubiquitin expressed in Alzheimer’s disease causes neuronal death. FASEB J (2001) 15:2680–8. 10.1096/fj.01-0438com 11726544

[71] Fischer DF de Vos RAI van Dijk R De Vrij FMS Proper EA Sonnemans MAF Disease-specific accumulation of mutant ubiquitin as a marker for proteasomal dysfunction in the brain. FASEB J (2003) 17:2014–24. 10.1096/fj.03-0205com 14597671

[72] de Pril R Fischer DF Maat-Schieman MLC Hobo B de Vos RAI Brunt ER Accumulation of aberrant ubiquitin induces aggregate formation and cell death in polyglutamine diseases. Hum Mol Genet (2004) 13:1803–13. 10.1093/hmg/ddh188 15198995

[73] Gentier RJ van Leeuwen FW. Misframed ubiquitin and impaired protein quality control: an early event in Alzheimer’s disease. Front Mol Neurosci (2015) 8:47. 10.3389/fnmol.2015.00047 26388726 PMC4557111

[74] Krutauz D Reis N Nakasone MA Siman P Zhang D Kirkpatrick DS Extended ubiquitin species are protein-based DUB inhibitors. Nat Chem Biol (2014) 10:664–70. 10.1038/nchembio.1574 24997605 PMC4466224

[75] Braun RJ Sommer C Leibiger C Gentier RJG Dumit VI Paduch K Accumulation of basic amino acids at mitochondria dictates the cytotoxicity of aberrant ubiquitin. Cell Rep (2015) 10:1557–71. 10.1016/j.celrep.2015.02.009 25753421 PMC4407011

[76] Tan Z Sun X Hou F-S Oh H-W Hilgenberg LGW Hol EM Mutant ubiquitin found in Alzheimer’s disease causes neuritic beading of mitochondria in association with neuronal degeneration. Cell Death Differ (2007) 14:1721–32. 10.1038/sj.cdd.4402180 17571083 PMC3258508

[77] Fischer DF van Dijk R van Tijn P Hobo B Verhage MC van der Schors RC Long-term proteasome dysfunction in the mouse brain by expression of aberrant ubiquitin. Neurobiol Aging (2009) 30:847–63. 10.1016/j.neurobiolaging.2008.06.009 18760506

[78] Irmler M Gentier RJG Dennissen FJA Schulz H Bolle I Hölter SM Long-term proteasomal inhibition in transgenic mice by UBB^+1^ expression results in dysfunction of central respiration control reminiscent of brainstem neuropathology in Alzheimer patients. Acta Neuropathol (2012) 124:187–97. 10.1007/s00401-012-1003-7 22730000 PMC3400757

[79] Schipanski A Oberhauser F Neumann M Lange S Szalay B Krasemann S Lectin OS-9 delivers mutant neuroserpin to endoplasmic reticulum associated degradation in familial encephalopathy with neuroserpin inclusion bodies. Neurobiol Aging (2014) 35:2394–403. 10.1016/j.neurobiolaging.2014.04.002 24795221

[80] Tank EMH True HL. Disease-associated mutant ubiquitin causes proteasomal impairment and enhances the toxicity of protein aggregates. PLoS Genet (2009) 5:e1000382. 10.1371/journal.pgen.1000382 19214209 PMC2633047

[81] Lam YA Pickart CM Alban A Landon M Jamieson C Ramage R Inhibition of the ubiquitin-proteasome system in Alzheimer’s disease. Proc Natl Acad Sci U S A (2000) 97:9902–6. 10.1073/pnas.170173897 10944193 PMC27620

[82] Lindsten K De Vrij FMS Verhoef LGGC Fischer DF van Leeuwen FW Hol EM Mutant ubiquitin found in neurodegenerative disorders is a ubiquitin fusion degradation substrate that blocks proteasomal degradation. J Cell Biol (2002) 157:417–27. 10.1083/jcb.200111034 11980917 PMC2173284

[83] van Tijn P De Vrij FMS Schuurman KG Dantuma NP Fischer DF van Leeuwen FW Dose-dependent inhibition of proteasome activity by a mutant ubiquitin associated with neurodegenerative disease. J Cell Sci (2007) 120:1615–23. 10.1242/jcs.03438 17405812

[84] Verhoef LGGC Heinen C Selivanova A Halff EF Salomons FA Dantuma NP. Minimal length requirement for proteasomal degradation of ubiquitin-dependent substrates. FASEB J (2009) 23:123–33. 10.1096/fj.08-115055 18796559

[85] van Tijn P Verhage MC Hobo B van Leeuwen FW Fischer DF. Low levels of mutant ubiquitin are degraded by the proteasome in vivo. J Neurosci Res (2010) 88:2325–37. 10.1002/jnr.22396 20336771

[86] Hope AD de Silva R Fischer DF Hol EM van Leeuwen FW Lees AJ. Alzheimer’s associated variant ubiquitin causes inhibition of the 26S proteasome and chaperone expression. J Neurochem (2003) 86:394–404. 10.1046/j.1471-4159.2003.01844.x 12871580

[87] van Tijn P Dennissen FJA Gentier RJG Hobo B Hermes D Steinbusch HWM Mutant ubiquitin decreases amyloid β plaque formation in a transgenic mouse model of Alzheimer’s disease. Neurochem Int (2012) 61:739–48. 10.1016/j.neuint.2012.07.007 22797007

[88] Lee J-G Takahama S Zhang G Tomarev SI Ye Y. Unconventional secretion of misfolded proteins promotes adaptation to proteasome dysfunction in mammalian cells. Nat Cell Biol (2016) 18:765–76. 10.1038/ncb3372 27295555 PMC10701763

[89] Komander D Rape M. The ubiquitin code. Annu Rev Biochem (2012) 81:203–29. 10.1146/annurev-biochem-060310-170328 22524316

[90] Yau R Rape M. The increasing complexity of the ubiquitin code. Nat Cell Biol (2016) 18:579–86. 10.1038/ncb3358 27230526

[91] Kwon YT Ciechanover A. The ubiquitin code in the ubiquitin-proteasome system and autophagy. Trends Biochem Sci (2017) 42:873–86. 10.1016/j.tibs.2017.09.002 28947091

[92] Steele JC Caparros-Lefebvre D Lees AJ Sacks OW. Progressive supranuclear palsy and its relation to pacific foci of the parkinsonism-dementia complex and Guadeloupean parkinsonism. Parkinsonism Relat Disord (2002) 9:39–54. 10.1016/S1353-8020(02)00043-3 12217621

[93] Dickson DW. Parkinson’s disease and parkinsonism: neuropathology. Cold Spring Harb Perspect Med (2012) 2:a009258. 10.1101/cshperspect.a009258 22908195 PMC3405828

[94] Jansen AHP Reits EAJ Hol EM. The ubiquitin proteasome system in glia and its role in neurodegenerative diseases. Front Mol Neurosci (2014) 7:73. 10.3389/fnmol.2014.00073 25152710 PMC4126450

[95] Choi K Park J Lee J Han EC Choi C Mutant ubiquitin attenuates interleukin-1β- and tumor necrosis factor-α-induced pro-inflammatory signaling in human astrocytic cells. PLoS One (2013) 8:e67891 10.1371/journal.pone.0067891 23844119 PMC3700915

[96] Yim N Ryu S-W Han EC Yoon J Choi K Choi C. Mutant ubiquitin UBB+1 induces mitochondrial fusion by destabilizing mitochondrial fission-specific proteins and confers resistance to oxidative stress-induced cell death in astrocytic cells. PLoS One (2014) 9:e99937. 10.1371/journal.pone.0099937 24941066 PMC4062464

[97] Lowe J McDermott H Landon M Mayer RJ Wilkinson KD. Ubiquitin carboxyl-terminal hydrolase (PGP 9.5) is selectively present in ubiquitinated inclusion bodies characteristic of human neurodegenerative diseases. J Pathol (1990) 161:153–60. 10.1002/path.1711610210 2166150

[98] Zouambia M Fischer DF Hobo B de Vos RAI Hol EM Varndell IM Proteasome subunit proteins and neuropathology in tauopathies and synucleinopathies: consequences for proteomic analyses. Proteomics (2008) 8:1221–36. 10.1002/pmic.200700679 18283660

[99] Leroy E Boyer R Auburger G Leube B Ulm G Mezey E The ubiquitin pathway in Parkinson’s disease. Nature (1998) 395:451–2. 10.1038/26652 9774100

[100] Bilguvar K Tyagi NK Ozkara C Tuysuz B Bakircioglu M Choi M Recessive loss of function of the neuronal ubiquitin hydrolase UCHL1 leads to early-onset progressive neurodegeneration. Proc Natl Acad Sci U S A (2013) 110:3489–94. 10.1073/pnas.1222732110 23359680 PMC3587195

[101] Saigoh K Wang YL Suh JG Yamanishi T Sakai Y Kiyosawa H Intragenic deletion in the gene encoding ubiquitin carboxy-terminal hydrolase in gad mice. Nat Genet (1999) 23:47–51. 10.1038/12647 10471497

[102] Tashiro Y Urushitani M Inoue H Koike M Uchiyama Y Komatsu M Motor neuron-specific disruption of proteasomes, but not autophagy, replicates amyotrophic lateral sclerosis. J Biol Chem (2012) 287:42984–94. 10.1074/jbc.M112.417600 23095749 PMC3522293

[103] Przedborski S. The two-century journey of Parkinson disease research. Nat Rev Neurosci (2017) 18:251–9. 10.1038/nrn.2017.25 28303016

[104] McNaught KSP Perl DP Brownell A-L Olanow CW. Systemic exposure to proteasome inhibitors causes a progressive model of Parkinson’s disease. Ann Neurol (2004) 56:149–62. 10.1002/ana.20186 15236415

[105] Kaushik S Cuervo AM Proteostasis and aging. Nat Med (2015) 21:1406–15. 10.1038/nm.4001 26646497

[106] Labbadia J Morimoto RI. The biology of proteostasis in aging and disease. Annu Rev Biochem (2015) 84:435–64. 10.1146/annurev-biochem-060614-033955 25784053 PMC4539002

[107] Klaips CL Jayaraj GG Hartl FU Pathways of cellular proteostasis in aging and disease. J Cell Biol (2018) 217:51–63. 10.1083/jcb.201709072 29127110 PMC5748993

[108] Hartl FU Bracher A Hayer-Hartl M. Molecular chaperones in protein folding and proteostasis. Nature (2011) 475:324–32. 10.1038/nature10317 21776078

[109] Ciechanover A Kwon YT Protein quality control by molecular chaperones in neurodegeneration. Front Neurosci (2017) 11:2491 10.3389/fnins.2017.00185 PMC5382173 28428740

[110] Kopito RR. Aggresomes, inclusion bodies and protein aggregation. Trends Cell Biol (2000) 10:524–30. 10.1016/S0962-8924(00)01852-3 11121744

[111] Lee B-H Lee MJ Park S Oh D-C Elsasser S Chen P-C Enhancement of proteasome activity by a small-molecule inhibitor of USP14. Nature (2010) 467:179–84. 10.1038/nature09299 20829789 PMC2939003

[112] Myeku N Clelland CL Emrani S Kukushkin NV Yu WH Goldberg AL Tau-driven 26S proteasome impairment and cognitive dysfunction can be prevented early in disease by activating cAMP-PKA signaling. Nat Med (2016) 22:46–53. 10.1038/nm.4011 26692334 PMC4787271

[113] Myeku N Duff KE. Targeting the 26S proteasome to protect against proteotoxic diseases. Trends Mol Med (2018) 24:18–29. 10.1016/j.molmed.2017.11.006 29233753 PMC5905406

[114] Ciechanover A Kwon YT. Degradation of misfolded proteins in neurodegenerative diseases: therapeutic targets and strategies. Exp Mol Med (2015) 47:e147. 10.1038/emm.2014.117 25766616 PMC4351408

[115] Hernebring M Brolén G Aguilaniu H Semb H Nyström T. Elimination of damaged proteins during differentiation of embryonic stem cells. Proc Natl Acad Sci U S A (2006) 103:7700–5. 10.1073/pnas.0510944103 16672370 PMC1472508

[116] Bohnert KA Kenyon C. A lysosomal switch triggers proteostasis renewal in the immortal *C. elegans* germ lineage. Nature (2017) 551:629–33. 10.1038/nature24620 29168500 PMC5936623

[117] Melentijevic I Toth ML Arnold ML Guasp RJ Harinath G Nguyen KC *C. elegans* neurons jettison protein aggregates and mitochondria under neurotoxic stress. Nature (2017) 542:367–71. 10.1038/nature21362 28178240 PMC5336134

[118] Kuzuhara S Kokubo Y Sasaki R Narita Y Yabana T Hasegawa M Familial amyotrophic lateral sclerosis and parkinsonism-dementia complex of the Kii Peninsula of Japan: clinical and neuropathological study and tau analysis. Ann Neurol (2001) 49:501–11. 10.1002/ana.100 11310628

[119] Kuzuhara S Kokubo Y. Atypical parkinsonism of Japan: amyotrophic lateral sclerosis-parkinsonism-dementia complex of the Kii peninsula of Japan (Muro disease): an update. Mov Disord (2005) 20(Suppl 12):S108–13. 10.1002/mds.20548 16092099

[120] Mimuro M Yoshida M Kuzuhara S Kokubo Y. Amyotrophic lateral sclerosis and parkinsonism-dementia complex of the Hohara focus of the Kii Peninsula: a multiple proteinopathy? Neuropathology (2018) 38:98–107. 10.1111/neup.12434 29063640

[121] Gajdusek DC Salazar AM. Amyotrophic lateral sclerosis and parkinsonian syndromes in high incidence among the Auyu and Jakai people of West New Guinea. Neurology (1982) 32:107–26. 10.1212/WNL.32.8.919-b 7198738

[122] Okumiya K Wada T Fujisawa M Ishine M Garcia Del Saz E Hirata Y Amyotrophic lateral sclerosis and parkinsonism in Papua, Indonesia: 2001–2012 survey results. BMJ Open (2014) 4:e004353. 10.1136/bmjopen-2013-004353 24740977 PMC3996815

